# Effect of Honey and Aqueous Garlic Extracts against Short-Term Exposure of Cigarette Tobacco Smoking in Mice: Histopathological and Biochemical Investigations

**DOI:** 10.1155/2024/5539447

**Published:** 2024-02-19

**Authors:** Ziad Shraideh, Darwish Badran, Ahmed Alzbeede

**Affiliations:** ^1^Department of Biological Sciences, School of Science, The University of Jordan, Amman 11942, Jordan; ^2^Department of Anatomy and Histology, Faculty of Medicine, The University of Jordan, and Ibn Sina University for Medical Sciences, Amman, Jordan; ^3^Department of Medical Laboratory Science, College of Science, Komar University of Science and Technology, Sulaymaniyah 46001, Kurdistan Region, Iraq

## Abstract

It is well known that cigarette smoking adversely affects human health and induces oxidative stress in most vital organs. This study aims to assess the biochemical, histological, and ultrastructural values of honey and garlic extracts in ameliorating the effects of short-term exposure to cigarette smoke in mice. Forty-eight mice were randomly divided into six equal groups: group I was exposed to fresh air only, group II was exposed to cigarette smoke, group III was given 0.2 ml of honey extract, group IV was exposed to cigarette smoke and was given 0.2 ml of honey extract, group V was given 0.2 ml of garlic extract, and group VI was exposed to cigarette smoke and was given 0.2 ml of aqueous garlic extract. These exposures were repeated daily for 21 consecutive days among the treated groups. By the end of the third week, the animals were euthanized by physical cervical dislocation. Blood was taken for biochemical study, and the selected organs of the liver, kidney, and jejunum were processed for histological and ultrastructural studies. The biochemical results showed that short-term exposure of experimental mice to cigarette smoking did not alter the liver function tests except for decreasing the albumin level. Moreover, cigarette smoking elevates the concentration of carbonyl protein content and cystatin C. Histologically, the use of honey and garlic showed good protection to the liver, kidney, and jejunum, which was proved by transmission electron microscopy, in addition to lowering the oxidative stress biomarkers. In conclusion, using honey and/or garlic helps protect the liver, kidney, and jejunum against the hazardous effects of cigarette smoke.

## 1. Introduction

Tobacco smoking through cigarettes or waterpipes contains multiple toxicants such as transition metals, carbon monoxide, aldehydes, nicotine, nitrosamine, and solid particulate matter [1]. The gaseous phase of cigarette smoke and the aqueous phase of cigarette tar contain significant amounts of free radicals with reactive oxygen and nitrogen species which eventually promote the production of hydroxyl radicals [2]. These chemical toxicants induce oxidative stress, vasoconstriction, inflammation, DNA damage, and adverse epigenetic regulation that contribute to organ dysfunction associated with adverse clinical outcomes [1–3]. Furthermore, prolonged secondhand exposure to tobacco smoking could progress into respiratory and cardiovascular risks in parallel with chronic obstructive pulmonary diseases [4].

Natural antioxidants are considered excellent therapeutic remedies to decrease oxidative stress by direct scavenging of reactive oxygen and nitrogen species that could affect human health [5]. Antioxidants are chemicals that are able to lower the activity of free radicals, therefore attenuating the risk of oxidative stress, and preserve the cellular components from alterations or damage [6].

The aqueous extracts of garlic and honey contain significant amounts of several phytochemicals potentially beneficial for human health [7, 8]. Experimental studies have shown that garlic extracts containing sulfur-containing bioactive compounds, such as alliin, allicin, e-ajoene, z-ajoene, 2-vinyl-4h-1,3-dithiin, diallyl sulfide, diallyl disulfide, diallyl trisulfide, and allyl methyl sulfide, have antibacterial, antihypertensive, cardioprotective, antilipidemic, anticarcinogenic, immunostimulant, and hypoglycemic properties [9, 10]. Honey possesses therapeutic properties attributed to its substantial quantity of flavonoids (chrysin, galangin, and quercetin) and phenolic acids (caffeic acid, gallic acid, and p-coumaric acid), which contribute to its antioxidant and anti-inflammatory effects [8]. It exhibits antibacterial and anticancer characteristics, lowers glucose levels, and protects the cardiovascular, neurological, respiratory, and gastrointestinal systems [11, 12].

This work aims to assess the ameliorative effect of the aqueous extracts of garlic and honey against secondhand exposure to cigarette tobacco smoking using histopathological and clinical chemistry parameters to compare among the experimental groups of albino mice.

## 2. Materials and Methods

### 2.1. Preparation of Aqueous Garlic and Honey Extract

Bee honey and garlic were freshly purchased from the local market. Both aqueous garlic and honey extracts were prepared using the procedure described by Waheeb and Ali [13].

### 2.2. Animal Grouping and Dose Administration

This study was operated according to the instructions of the Scientific Committee in the School of Science at the University of Jordan in terms of animal handling and care. These instructions are consistent with the NIH Guide for the Care and Use of Laboratory Animals 2011 [14]. Male albino BALB/c mice were purchased from the Animal Household/University of Jordan. All adult males were about eight weeks old, weight 22 ± 3 g. The cigarettes: red labeled (L&M; 10 mg tar, 0.8 mg nicotine, and 10 mg CO) marked cigarettes were purchased from local markets (Philip Morris, Jordan).

Forty-eight adult male mice were randomly divided into 6 experimental groups: each group contains 8 mice. Group I (control): mice were exposed to fresh air only. Group II (SMK): mice were exposed to cigarette smoking. Group III (honey): mice were given 0.2 ml of prepared aqueous honey extract through oral gavage. Group IV (SMK + honey): mice were exposed to cigarette smoking and given 0.2 ml of prepared aqueous honey extract through oral gavage. Group V (garlic): mice were given 0.2 ml of prepared aqueous garlic extract through oral gavage. Group VI (SMK + garlic): mice were exposed to cigarette smoking and given 0.2 ml of prepared aqueous garlic extract through oral gavage. These exposures were repeated daily for 21 consecutive days among the treated groups.

The process of cigarette smoking exposure was explained by Alzbeede et al. [15] using a modified smoking machine described by Shraideh et al. [16].

### 2.3. Tissue Separation and Processing

By the end of the 3rd week, the mice were euthanized by physical cervical dislocation. Blood was withdrawn through inserting a capillary tube into the venous orbital plexus, and serum was separated from the blood (clotting time 60 minutes in room temperature under dark conditions, followed by centrifugation 3800 rpm-10 minutes) for biochemical analysis.

Mice were dissected to obtain the liver, kidney, and jejunum. These organs were washed with phosphate buffered saline (PBS) (0.01 M-pH 7.2) and fixed in 10% formal saline for light microscopy or 2.5% glutaraldehyde in PBS for electron microscopy techniques. Steps of light and transmission electron microscopy were described by Alzbeede et al. [15]. Organs were dehydrated with ascending grades of ethanol, cleared with xylene, and impregnated and embedded in melted paraffin wax for the light microscopy technique. Then, these samples were sectioned at 5 *μ*m thickness, stained with hematoxylin and eosin stains, mounted by DPX, and examined using light microscopy.

Besides, small pieces of the selected organs were postfixed with 1% osmium tetroxide, dehydrated with ethanol, cleared by propylene oxide, and infiltrated with 1 : 1 propylene oxide:resin, and embedded in epoxy resin. These specimens were sectioned using an ultramicrotome, then mounted on copper grids and stained with uranyl acetate and counter-stained with lead citrate, and then, the sections were then ready to be examined by transmission electron microscopy.

### 2.4. Biochemical Testing

The separated serum was used to estimate some biochemical markers such as liver function tests, e.g., alanine aminotransferase (ALT), aspartate aminotransferase (AST), lactate dehydrogenase (LDH), gamma-glutamyl transferase (*γ*GT), total protein, albumin, globulin, total bilirubin, and kidney function tests, e.g., creatinine, urea, and cystatin C. Besides, quantitative measurements of carbonyl protein content and malonaldehyde (MDA) were performed to evaluate protein and lipid peroxidation, respectively. All biochemical tests listed above were completed using a fully automated biochemical analyzers (HumaStar 600, Human Diagnostics Worldwide, Germany) except cystatin C and carbonyl protein contents that were quantified according to the instructions provided by the kit manufacturer (ab119590, Abcam, UK) and (ab126287, Abcam, UK), respectively, using a 96-well plate reader (Synergy HTX, BioTeK, USA). The evaluation of lipid peroxidation was performed by the determination of malondialdehyde (MDA) concentration in plasma samples, according to Lima et al. [17]. A serum sample of 250 *μ*l was mixed with 500 *μ*l of 0.6% 2-thiobarbaturic acid (TBA) (T5500, Sigma Aldrich, USA).

### 2.5. Statistical Analysis

The results were tabulated in GraphPad Prism version 8.2.1, IBM, USA, for statistical analysis. All data of the treatment groups for each biochemical test were analyzed by one-way ANOVA, followed by a Tukey post hoc comparison test and considering *P* value <0.05.

## 3. Results

### 3.1. Biochemical Investigation of Liver, Kidney, and Oxidative Stress Biomarkers

Mice exposed to cigarette smoke showed a significant decrease in albumin concentration compared to the control, honey, and garlic groups at *P* < 0.01 (0.0073), *P* < 0.01 (0.0016), and *P* < 0.05 (0.0158), respectively (Table 1). However, treatments with honey or garlic were in parallel with exposure to cigarette smoke did not show any significant differences with control, honey, and garlic groups at *P* < 0.05 (Table 1). The AST activity result revealed that the smoking group has slightly increased with a significant difference against the garlic group only *P* < 0.05 (0.0353) (Table 1). Otherwise, there are no significant differences among the experimental groups (Table 1). The concentrations of total protein, globulins, total bilirubin, and the enzymatic activity of ALT and *γ*GT did not show any significant differences among the treated groups at *P* < 0.05 (Table 1).

Kidney function tests illustrated that the concentrations of creatinine and urea did not show any significant differences among the study groups (Table 1). However, the cystatin C levels of the SMK group showed significant results compared to honey and garlic groups at *P* < 0.01 (0.0073) and *P* < 0.01 (0.0016), respectively, but this slight increase in cystatin C level was not significant against the control group at *P* < 0.05 (Table 1).

**Figure 1:**
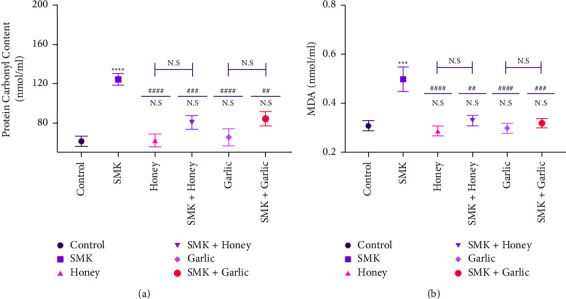
The concentration of two oxidative stress markers in the serum of experimental mice in this study was presented by a mean ± SEM plotting graph. (a) Concentration of protein carbonyl content in nmol/ml; (b) concentration of malondialdehyde in nmol/ml; significancy versus control group represented by (^*∗*^ asterisks); significancy versus control group represented by (# hashtag). The blue-capped lines represent the significance between the intergroups and could be assigned with $, $$, $$$, N.S. single, double, triple, and quadruple signs indicate *P* < 0.05, *P* < 0.01, *P* < 0.001, and *P* < 0.0001, respectively, and N.S represents no significance when *P* ≥ 0.05.

Carbonyl protein content is illustrated in Figure 1(a) and the SMK group has significant differences with all other experimental groups at different *P* values (*P* < 0.0001 versus control, honey, and garlic groups, respectively) and (*P*=0.0006 versus SMK + honey; *P*=0.0021 versus SMK + garlic group). SMK + honey group did not show any significant differences with the control or honey group. The same thing was revealed with the garlic group that did not show any significant differences with the control and garlic group at *P* < 0.05.

Lipid peroxidation level was estimated depending on malonaldehyde quantification (Figure 1(b)). It showed that the SMK group has a significantly higher concentration than the control, honey, and garlic groups at *P* < 0.0001. Moreover, the SMK group showed significant differences with SMK + honey (*P*=0.0006) and SMK + garlic (*P*=0.0021). The SMK + honey group did not show any significant differences with the control or honey group. The same thing was revealed with the garlic group that did not show any significant differences with the control and garlic group at *P* < 0.05.

### 3.2. Histopathological Examination of Hepatic Cells Using Light Microscopy

**Figure 2:**
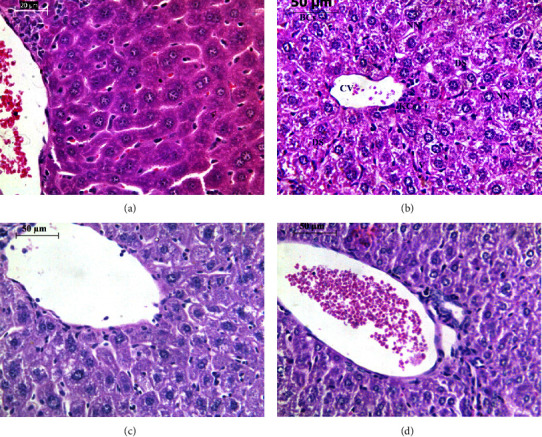
Images of liver sections for the experimental groups in this study stained with hematoxylin and eosin: (a) control group, (b) exposure to cigarette smoking, (c) exposure to cigarette smoking with parallel treatment to honey, and (d) exposure to cigarette smoking with parallel treatment to aqueous garlic extract. These photos were taken with a colored digital camera (Leica EC3, Switzerland) attached to a Leica inverted light microscope (Leica microsystems, Germany), and they were manually examined using computer software (Leica application suite LAS EZ version 1.8.0, Leica microsystems, Switzerland).

Sections of the liver of the control group had shown histologically normal liver architecture, containing normal hepatocytes and sinusoids, a central vein, and many binucleated hepatocytes (Figure 2(a)). The histological sections of the liver of the CS group showed that Kupffer cells became prominent and increased in number and sinusoids are dilated (Figure 2(b)). Hepatocytes showed a high degree of vacuolization, chromatin of nuclei was condensed into peripheral clumps and congested blood vessels (Figure 2(b)). Following treatment with honey, the liver of mice of this group showed signs of partial recovery (Figure 2(c)). The treatment resulted in improvement in hepatocyte architecture, less vacuolization, and less infiltration of immune cells and Kupffer cells. In addition, treatment with garlic showed a certain degree of protection against CS damage (Figure 2(d)). The hepatocytes are still showing a lesser degree of vacuolization and less degree of infiltration of Kupffer cells in comparison to the CS group (Figure 2(d)).

### 3.3. Histopathological Examination of Nephrons Using Light Microscopy

**Figure 3:**
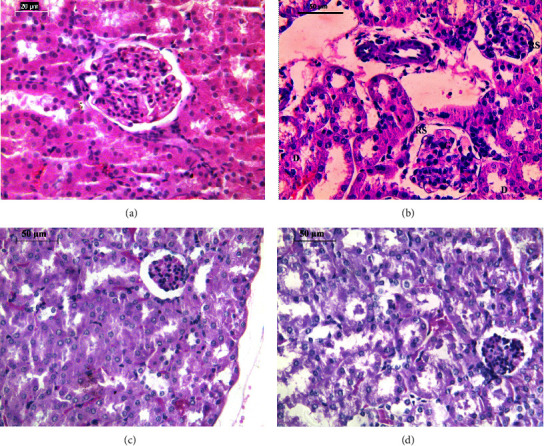
Images of kidney sections for the experimental groups in this study stained with hematoxylin and eosin: (a) control group, (b) exposure to cigarette smoking, (c) exposure to cigarette smoking with parallel treatment to honey, and (d) exposure to cigarette smoking with parallel treatment to aqueous garlic extract. These photos were taken with a colored digital camera (Leica EC3, Switzerland) attached to a Leica inverted light microscope (Leica microsystems, Germany), and they were manually examined using computer software (Leica application suite LAS EZ version 1.8.0, Leica microsystems, Switzerland).

The section of kidney samples from the control group is represented in Figure 3(a). It showed normal integrity of the renal cortex, with a normal renal corpuscle characterized by glomeruli and Bowman's capsule surrounded by proximal and distal convoluted tubules (Figure 3(a)).

The kidney sections of mice exposed to cigarette smoking showed several abnormalities, including widening of the renal space, mild condensed glomeruli, heterochromatic nuclei, cell vacuolization, partially disrupted epithelia, or even desquamation of the tubular cells. Following treatment with honey, the kidney of mice of this group showed signs of partial prophylaxis by demonstrating improvements in renal architecture, condensation of the glomerulus, no cellular vacuolization, and normal nuclei of tubular cells (Figure 3(c)). Treatment with garlic showed a certain degree of protection of the kidney against CS damage (Figure 3(d)). There is still less glomeruli condensation and partial disruption of the tubular epithelium (Figure 3(d)).

### 3.4. Histopathological Examination of the Jejunum Using Light Microscopy

**Figure 4:**
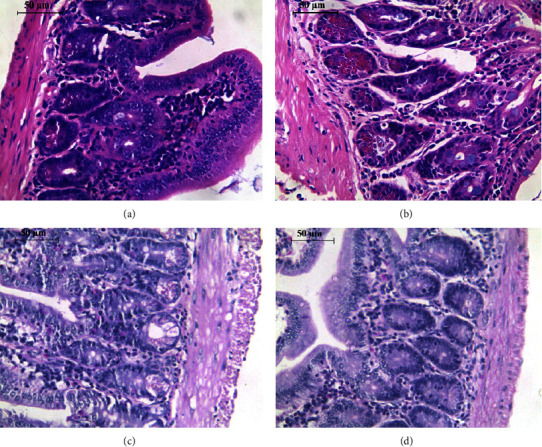
Images of jejunum sections for the experimental groups in this study stained with hematoxylin and eosin: (a) control group, (b) exposure to cigarette smoking, (c) exposure to cigarette smoking with parallel treatment to honey, and (d) exposure to cigarette smoking with parallel treatment to aqueous garlic extract. These photos were taken with a colored digital camera (Leica EC3, Switzerland) attached to a Leica inverted light microscope (Leica microsystems, Germany), and they were manually examined using computer software (Leica application suite LAS EZ version 1.8.0, Leica microsystems, Switzerland).

The cross-section of the jejunum of the control mice revealed the typical architecture of the epithelial layer of the small intestine associated with normal enterocytes, goblet cells, and intestinal glands with Paneth cells (Figure 4(a)). Exposure to cigarette smoking showed several abnormalities as enterocytes appeared with heterochromatic nuclei and cellular vacuolization, in addition to increasing the number of Paneth cells and infiltration of macrophages in the lamina propria (Figure 4(b)). Following treatment with honey, the jejunum of mice of this group showed good protection against CS; there is an improvement in intestine architecture, no cellular vacuolization, and normal nuclei of enterocytes (Figure 4(c)). Moreover, treatment with garlic ameliorated the effect of CS; the intestinal architecture appears normal, cellular vacuolization is less, and normal nuclei of enterocytes (Figure 4(d)).

### 3.5. Ultrastructural Observation of Hepatic Cells Using Transmission Electron Microscopy

**Figure 5:**
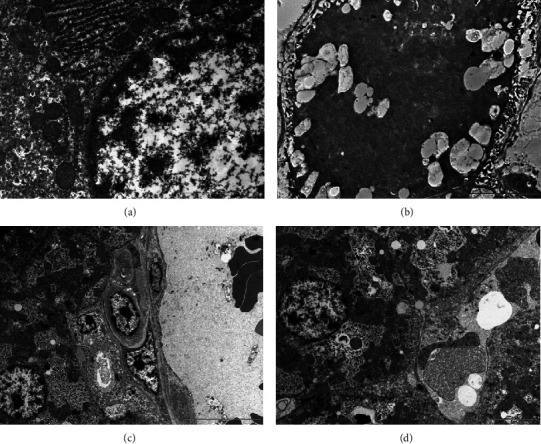
Ultrathin sections of 70 nm from livers of different experimental groups. (a) Control group, (b) cigarette smoking showing many vacuoles (arrows), (c) cigarette smoking + honey, and (d) cigarette smoking + garlic. Sections were stained with uranyl acetate and counter-stained with lead citrate; the scale bars presented in the lower right corner of the images; these images were captured using a transmission electron microscope (Morgagni 268, FEI Philips, Netherlands), monitored through computer software (Morgagni 268D, version 2.21, Netherlands).

In ultrathin sections of the liver from the control experimental mice, normal hepatocytes were seen (Figure 5(a)). These cells had characteristically large, round, predominantly euchromatic nuclei. There were several obvious, clearly cristae-containing mitochondria in the cytoplasm, as well as many profiles of rough endoplasmic reticulum (Figure 5(a)). In contrast, hepatocytes of mice that were exposed to cigarette smoking displayed polymorphic mitochondria and mild deterioration in the cristae and matrix; the nuclei contained some chromatin condensation next to the inner margins of the nuclear envelope; the nuclear envelope appeared irregular in shape; a rough endoplasmic reticulum appeared scattered with dilated cisternae; and the cytosol content was distorted with the presence of some vacuolization (Figure 5(b)).

Comparing portions of hepatocytes treated with honey to hepatocytes that had been exposed to smoke, the outcomes were better. It revealed decreased cellular blebbing, preserved normal mitochondrial shape and size, and increased euchromatic chromatin proportions in the nuclei (Figure 5(c)). Ultrastructural examination of the liver in mice receiving the aqueous garlic extract showed good protection against CS, as seen in Figure 5(d). The mitochondria appear to have proper cristae, and the cells did not exhibit vacuolization or multilamellar structures. In addition, chromatin is less condensed in nuclei (Figure 5(d)).

### 3.6. Ultrastructural Observation of Nephron Using Transmission Electron Microscopy

**Figure 6:**
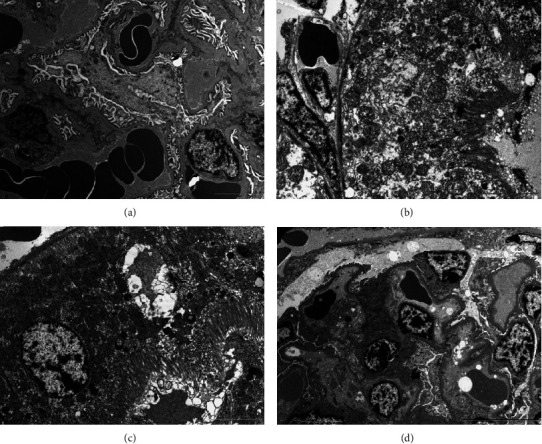
Ultrathin sections of 70 nm from kidney of different experimental groups. (a) Control group, (b) cigarette smoking, (c) cigarette smoking + honey, and (d) cigarette smoking + garlic. Sections were stained with uranyl acetate and counter-stained with lead citrate; the scale bars presented in the lower right corner of the images; these images were captured using transmission electron microscope (Morgagni 268, FEI Philips, Netherlands), monitored through computer software (Morgagni 268D, version 2.21, Netherlands). The ultrathin sections were photographed at 60 kilovolts at different low power magnifications.

The ultrathin section from the cortical part of the nephron in the control group showed normal integrity of the renal cortex, with normal glomeruli surrounded by proximal and distal convoluted tubules (Figure 6(a)). The examined ultrathin sections of cigarette smoke exposed showed a high degree of cytoplasmic vacuolization and polymorphic, swollen mitochondria. There was also extruded material in the renal space and partially disrupted capillary endothelium (Figure 6(b)).

Following treatment with honey, the kidney of mice of this group showed good protection against CS (Figure 6(c)). The treatment resulted in improvement in kidney ultrastructure, a smaller number of vacuoles and lysosomes in the cytoplasm, and mitochondria looking with normal cristae. Moreover, nuclei have less condensed chromatin surrounded by a nuclear envelope with an intact appearance (Figure 6(c)). Following treatment with garlic, the kidney of mice of this group showed good protection against CS (Figure 6(d)). Renal corpuscle has normal architecture, no vacuolization of cells, normal filtration membrane, and intact endothelium. Podocytes have euchromatic nuclei (Figure 6(d)).

### 3.7. Ultrastructural Observation of the Jejunum Using Transmission Electron Microscopy

**Figure 7:**
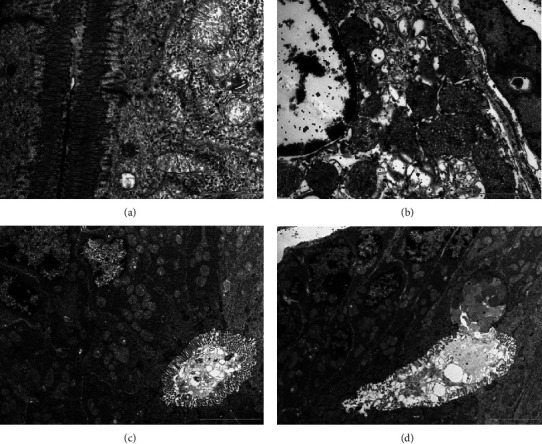
Ultrathin sections of 70 nm from jejunum of different experimental groups. (a) Control group, (b) cigarette smoking, (c) cigarette smoking + honey, and (d) cigarette smoking + garlic. Sections were stained with uranyl acetate and counter-stained with lead citrate; the scale bars presented in the lower right corner of the images; these images were captured using a transmission electron microscope (Morgagni 268, FEI Philips, Netherlands), monitored through computer software (Morgagni 268D, version 2.21, Netherlands). The ultrathin sections were photographed at 60 kilovolts at different low power magnifications.

The jejunum of the control group showed normal architecture: enterocytes with densely packed apical microvilli, mitochondria with normal cristae, and intercellular junctions; all cellular organelles were enclosed within well intact and resembled plasma membranes with deformities. The ultrastructural examination of the jejunum in mice exposed to cigarette smoking showed to exhibit edema, minor deformation, and dilated cell membranes that resembled blebs (Figure 7(b)). They have excessively thick bodies with tiny, lipid-containing vacuoles dispersed throughout the cytoplasm, spherical mitochondria with damaged cristae, and uneven, shortened microvilli baselines (Figure 7(b)). Due to cellular swelling, the enterocytes in this group partially lost their sinuosity, a sign of moderate to severe structural damage (Figure 7(b)).

Following treatment with honey, the jejunum of mice of this group showed good protection against CS (Figure 7(c)). The treatment resulted in improvements in enterocyte ultrastructure. No cellular vacuolization and euchromatic nuclei of enterocytes (Figure 7(c)). Following treatment with garlic, the jejunum of mice of this group showed good protection against CS (Figure 7(d)). The treatment resulted in improvements in intestine ultrastructure, no cellular vacuolization, and euchromatic nuclei of enterocytes. The goblet cells showed a normal appearance with extracellular secretions (Figure 7(d)).

## 4. Discussion

Despite recent declines in its prevalence, tobacco smoking remains among the primary causes of illness and early death globally [18]. Tobacco smoking in all its forms has harmful effects on active and passive smokers, leading to oxidative stress due to chemical constituents containing various free radicals [19]. The biochemical results of this study showed that short-term exposure of experimental mice to cigarette smoking did not alter the liver function tests except for a decrease in the albumin level. These results are inconsistent with those of Nemmar et al. that showed a significant increase in the enzymatic activity of ALT and *γ*GT by the action of passive inhaled cigarette smoking in mice [20].

The assumption of a decline in the albumin level caused by severe hepatic damage that could have a direct effect on albumin production or leakage of albumin through renal filtration were negligible since most markers of liver and kidney function tests were normal in this study. Moreover, according to a case-control study of patients with acute aortic dissection, hypoalbuminemia, smoking, and hypertension are strongly associated with this condition [21]. Another finding of this study is that short-term inhaled cigarette smoking affected the albumin and cystatin C concentrations in serum, which were corrected by oral administration of aqueous honey or garlic extract. In addition, aqueous extracts of honey or garlic given orally have antioxidative capabilities since they reduce the levels of lipid peroxidation and protein carboxylation biomarkers in the serum of the experimental mice used in this investigation.

Histologically, it is apparent that the use of honey and the aqueous solution of garlic had a protective effect on the tissues studied; hepatocytes appeared normal, kidney tissue showed almost complete recovery, and the enterocytes of the jejunum appeared normal. This is consistent with the biochemical findings.

Cigarette smoking caused abnormal physiological indices (such as reduced body weight, blood lipid levels, and food intake) that resulted in hepatocellular damage, abnormalities in the liver transcriptome during lipid metabolism, and disruption of the gut microbiota in mice [22–24]. Moreover, other findings showed the renal function of mice was negatively affected by exposure to cigarette smoking, which led to an acceleration of renal fibrosis and cystic development as well as a physiological reduction in glomerulus filtration rate [25, 26]. Exposure to cigarette smoke increases intestinal mucosal disruption, intestinal barrier dysfunction, oxidative stress, apoptosis, and tight junction component dysregulation [27–29]. It also causes mucosal inflammation with specific changes in Paneth cell granules [30].

In this study, parallel treatment with honey or aqueous garlic extract showed signs of partial recovery in hepatocyte architecture, slight improvement in cellular compartments in the cortex part of the kidney, and maintain the morphological structure of the intestinal mucosal layer against passive exposure to cigarette smoking.

It was explained that garlic extracts containing active compounds such as allicin, diallyl disulfide, S-allyl disulfide, and S-allylcysteine have antihypertensive, inhibiting the assembly and disassembly processes of the cytoskeleton, attenuate inflammation, and induce immunomodulatory effect [31, 32]. It was claimed that the phytoantioxidants content in garlic extract could neutralize the hepatotoxicity of administrated heavy metals like lead nitrate and lead acetate [33, 34]. Because the treatment of aqueous garlic extract was able to reverse these oxidant reactions, as well as improve renal function and histological damage, it is plausible that aqueous garlic extract shields the tissues from nicotine-induced oxidative damage [35]. Furthermore, both garlic oil and diallyl disulfide have shown promise as possible anti-inflammatory agents in the treatment of airway inflammation caused by cigarette smoking [36]. Aged red garlic extract inhibits cisplatin-induced cell death in human bronchial smooth muscle cells by boosting reduced glutathione concentration and decreasing reactive oxygen species production [37].

Several studies have shown the protective effect of honey on other body tissues. The administration of honey reduced the alveolar wall damage in mice brought on by cigarette smoking by diminishing the lumen of the alveolus that had undergone enlargement and infiltration of inflammatory cells [38]. When chrysin (a polyphenol available in honey) was administered intraperitoneally in various dosages, it prevented cigarette smoke-induced inflammation by decreasing the inflammatory cytokines and myeloperoxidase activity by lowering the phosphorylation of p38 and ERK [39, 40]. Honey protects rat testes from smoking-induced histological alterations and oxidative stress, according to several studies [41, 42]. Honey considerably decreased the harmful effects of cigarette smoking on testicular structures and oxidative stress by lowering lipid peroxidation and restoring the antioxidant system [43, 44].

## 5. Conclusion

This study proposes that the aqueous extract of honey and garlic may possess a mitigating effect on oxidative damage in the specific organs examined in this research, which was induced by short-term subacute secondhand (passive) exposure to cigarette tobacco smoke. Currently, smoking cessation is widely regarded as the most effective approach to mitigate the detrimental effects of inhaled toxins.

## Figures and Tables

**Table 1:** Effect of aqueous honey and garlic extracts on some liver and kidney function parameters in parallel with the inhalation toxicity induced by cigarette smoking (SMK).

Biochemical test	Treatment groups					
	Control	SMK	Honey	SMK + honey	Garlic	SMK + garlic
Total protein (g/dL)	5.68 ± 0.29	5.20 ± 0.32	5.53 ± 0.38	5.48 ± 0.32	5.87 ± 0.41	5.5 ± 0.33
Albumin (g/dL)	3.31 ± 0.16	2.51 ± 0.13^*∗∗*^	3.42 ± 0.17^##^	2.95 ± 0.15	3.25 ± 0.16^#^	2.91 ± 0.14
Globulins (g/dL)	2.36 ± 0.12	2.69 ± 0.17	2.21 ± 0.32	2.54 ± 0.11	2.62 ± 0.31	2.61 ± 0.14
Total bilirubin (mg/dL)	0.09 ± 0.01	0.09 ± 0.01	0.12 ± 0.01	0.10 ± 0.01	0.09 ± 0.01	0.09 ± 0.01
AST (U/L)	202 ± 12.12	237 ± 11.2	172 ± 20	218 ± 23	159 ± 22.4^#^	214 ± 12.8
ALT (U/L)	48.6 ± 1.72	58 ± 2.27	51 ± 3.5	49 ± 2.6	51.3 ± 3.2	54 ± 1.85
*γ*GT (U/L)	2.81 ± 0.32	2.84 ± 0.2	2.75 ± 0.5	2.7 ± 0.3	2.33 ± 0.36	2.48 ± 0.26
Creatinine (mg/dL)	0.30 ± 0.01	0.37 ± 0.05	0.30 ± 0.01	0.31 ± 0.01	0.33 ± 0.02	0.36 ± 0.01
Urea (mg/dL)	32.3 ± 0.7	41.2 ± 4.88	38.1 ± 1.44	39.9 ± 1.53	32.2 ± 2.81	3.4 ± 1.6
Cystatin C (ng/ml)	77.4 ± 4.41	90.6 ± 6.23	54.9 ± 3.30^##^	77.2 ± 4.63	54.5 ± 3.33^##^	81.5 ± 4.90

^*∗∗*^ *P* < 0.01, ^*∗*^*P* < 0.05 compared with the control group. ^##^*P* < 0.01, ^#^*P* < 0.05 compared with the SMK group. Alanine aminotransferase (ALT), aspartate aminotransferase (AST), lactate dehydrogenase (LDH), and gamma-glutamyl transferase (*γ*GT). Data values are represented in (mean ± SEM); *n* = 8.

## Data Availability

The majority of data are already illustrated in the result part. The raw data of this work would be available upon reasonable request.

## Additional Points

*Highlights*. (i) Short-term exposure to cigarette tobacco smoking induces biochemical changes and histopathological alterations in the liver, kidney, and jejunum in mice. (ii) Aqueous honey and garlic extracts have a prophylactic effect of reducing the cytotoxicity induced by cigarette smoking in the vital organs of this study. (iii) Aqueous honey and garlic extract could reduce protein carbonyl content and malondialdehyde concentration in the plasma of mice exposed to cigarette smoking.
